# Improved Detection of *Mycobacterium bovis* Infection in Bovine Lymph Node Tissue Using Immunomagnetic Separation (IMS)-Based Methods

**DOI:** 10.1371/journal.pone.0058374

**Published:** 2013-03-04

**Authors:** Linda D. Stewart, James McNair, Lyanne McCallan, Alan Gordon, Irene R. Grant

**Affiliations:** 1 Institute for Global Food Security, School of Biological Sciences, Queen's University Belfast, Belfast, Northern Ireland, United Kingdom; 2 Veterinary Sciences Division, Agri-Food and Biosciences Institute for Northern Ireland, Stormont, Belfast, Northern Ireland, United Kingdom; 3 Biometrics Division, Agri-Food and Biosciences Institute for Northern Ireland, Newforge Lane, Belfast, Northern Ireland, United Kingdom; University College Dublin, Ireland

## Abstract

Immunomagnetic separation (IMS) can selectively isolate and concentrate *Mycobacterium bovis* cells from lymph node tissue to facilitate subsequent detection by PCR (IMS-PCR) or culture (IMS-MGIT). This study describes application of these novel IMS-based methods to test for *M. bovis* in a survey of 280 bovine lymph nodes (206 visibly lesioned (VL), 74 non-visibly lesioned (NVL)) collected at slaughter as part of the Northern Ireland bovine TB eradication programme. Their performance was evaluated relative to culture. Overall, 174 (62.1%) lymph node samples tested positive by culture, 162 (57.8%) by IMS-PCR (targeting IS6110), and 191 (68.2%) by IMS-MGIT culture. Twelve (6.9%) of the 174 culture positive lymph node samples were not detected by either of the IMS-based methods. However, an additional 79 *M. bovis* positive lymph node samples (27 (13.1%) VL and 52 (70.3%) NVL) were detected by the IMS-based methods and not by culture. When low numbers of viable *M. bovis* are present in lymph nodes (e.g. in NVLs of skin test reactor cattle) decontamination prior to culture may adversely affect viability, leading to false negative culture results. In contrast, IMS specifically captures whole *M. bovis* cells (live, dead or potentially dormant) which are not subject to any deleterious treatment before detection by PCR or MGIT culture. During this study only 2.7% of NVL lymph nodes tested culture positive, whereas 70.3% of the same samples tested *M. bovis* positive by the IMS-based tests. Results clearly demonstrate that not only are the IMS-based methods more rapid but they have greater detection sensitivity than the culture approach currently used for the detection of *M. bovis* infection in cattle. Adoption of the IMS-based methods for lymph node testing would have the potential to improve *M. bovis* detection in clinical samples.

## Introduction

Bovine tuberculosis (bTB) is an infectious disease of cattle caused by the bacterium *Mycobacterium bovis*, a member of the *Mycobacterium tuberculosis* (MTB) complex. It is a significant animal health issue in countries including the United Kingdom and Ireland where its incidence has been broadly rising for 25 years. This affects cattle based agriculture impacting on profitability and trade. *M. bovis* has a wide host range [1] making eradication difficult and resulting in some animals becoming wildlife reservoirs of the disease [2], [3]. The current primary method of screening live cattle in the UK for bTB is the single intradermal comparative cervical tuberculin (SICCT) test, carried out using purified protein derivative (PPD) from *M. bovis* and *Mycobacterium avium,* according to Annex B of European Directive 63/432/EEC (as amended).

Under EU legislation, all skin test reactors are mandatorily slaughtered and tissues may be removed for disease confirmation by histopathology or mycobacterial culture. Culture of *M. bovis* is the current ‘gold standard’ for confirmation of disease diagnosis [4]. However, it can be problematic for a number of reasons which include: i) the precise selection of infected tissue for culture; ii) the paucibacillary nature of *M. bovis* in the tissues selected; iii) harsh decontamination procedures that are a prerequisite to culture which can significantly reduce *M. bovis* viability; and iv) the slow-growing nature of *M. bovis* resulting in an eight week time lag for confirmation of its presence or absence in clinical samples. The outcome is that mycobacterial culture is both time consuming and expensive, insensitive due to ii) and iii), and delays disease confirmation.

A major difficulty with diagnosing bTB is that there is no simple, sensitive test to determine when a bacterium is viable, other than by using culture methods. Low culture positive rates for skin test NVL reactors might be due to low numbers of bacteria in tissues and to the detrimental effects of decontamination with potentially low recovery of bacilli. In view of the low culture positive rate for *M. bovis* from reactors with no visible lesions (NVL), there is the possibility of a gross underestimation in the proportion of test reactor cattle with confirmed TB infection. This could, in turn, affect the way in which TB breakdowns are managed, since failure to detect visible lesions and to recover *M. bovis* may lead to a less stringent follow-up TB herd testing regime to regain officially TB free status. Tissues from test reactor animals can contain visible lesions (VL) or there may be no visible lesions (NVL) evident. In the United Kingdom, under EU legislation, all cattle routinely slaughtered for human consumption and not removed as SICTT reactors are routinely checked post mortem for the presence of tuberculous lesions. When suspect TB lesions are found in non-reactor animals during meat inspection at routine slaughter (designated “lesions at routine slaughter” in Northern Ireland or “slaughterhouse case” in Great Britain context) a sample of the affected tissue is also sent for TB culture. The protocol used for the selection of tissue samples for mycobacterial culture in the UK differs depending on, inter alia, the results of the herd level SICTT test, the history of the herd and animal, and the presence or absence of visible TB lesions at slaughter, i.e. not all reactors will have the same tissue samples examined. Indeed, many have no further sampling e.g. when disease previously confirmed in the herd. When lesions are present a small portion of infected tissue (<1 g) is prepared for culture (as well as histopathology), however, as confirmation rates of *M. bovis* infection in NVL samples by conventional culture are low, a larger sample of lymph node tissue (≤10 g) is cultured from NVL reactor animals.

The development of a rapid, more sensitive and specific screening test for *M. bovis* would allow veterinary authorities to quickly confirm bTB infection (and more often) in suspect herds. We recently reported the production of novel monoclonal antibodies and phage-display derived peptide ligands to *M. bovis* surface antigens and their evaluation for immunomagnetic separation (IMS) [5]. Tosylactivated Dynabeads dually coated with an IgM monoclonal antibody and a biotinylated 12– mer peptide binder achieved maximal capture of *M. bovis* from broth cultures and spiked lymph node samples. Preliminary studies determined that these binders were both specific and sensitive when applied to pure broth cultures of a range of commonly locally isolated spoligotypes of *M. bovis*, and that there was no significant cross-reactivity with any of the environmental *Mycobacterium* spp. tested or *M. bovis* BCG. In the UK, the TB culture method involves chemical decontamination of lymph node samples with oxalic acid, which is known to have an adverse effect on the viability of mycobacteria, in addition to the desired killing effect on non-mycobacterial contaminants [6]. IMS could potentially circumvent the need for chemical decontamination, by selectively capturing *M. bovis* cells and separating them from other bacteria in the samples, thereby retaining viability of *M. bovis* cells. Furthermore, IMS introduces specificity for *M. bovis* prior to IS6110 PCR and also removes sample matrix constituents that may inhibit the Taq enzyme used in the PCR reaction. Our original intention was to employ IMS in conjunction with a phage amplification assay to achieve rapid detection (within 48 h) of viable *M. bovis* cells in lymph node tissue, as has been reported previously for *M. avium* subsp. *paratuberculosis* [7], [8]. However, preliminary testing of naturally infected lymph nodes, prior to commencement of the larger scale testing reported here, yielded some unexpected results which meant that our proposed testing approach using an IMS-phage assay was not feasible. Therefore, the aim of the present study was to evaluate the performance of the recently developed and optimised IMS method (5), used in combination with IS6110 touchdown PCR and MGIT liquid culture, to detect *M. bovis* during a survey of naturally infected bovine lymph node tissues. IS6110 is a multiple copy insertion element originally thought to be exclusively found in the genome of MTB complex organisms [9], [10], [11]. However, it has recently been found in the genome of *Mycobacterium smegmatis* [12] and another environmental *Mycobacterium* sp. [13]. In light of the fact that the optimised IMS method introduces specificity for *M. bovis,* the fact that IS6110 may be detected from other mycobacterial species is immaterial in relation to IMS-PCR results. Results obtained with the IMS-based methods were compared with results obtained with the reference mycobacterial culture and direct PCR methods. The IMS-based methods were demonstrated to have greater *M. bovis* detection capability, particularly when testing non-visibly lesioned lymph nodes from reactor cattle.

## Materials and Methods

### Samples tested

A selection of 280 bovine tissue samples submitted routinely to the Statutory TB Laboratory, Agri-Food and Biosciences Institute for Northern Ireland (AFBI), Stormont, Belfast, were tested during this study; 240 were from skin test reactor animals (74 NVL and 166 VL) and 40 were from non-reactor animals with lesions detected at routine slaughter. Permission was given by Department of Agriculture and Rural Development (DARD) for Northern Ireland to test samples submitted under its TB eradication scheme during this study. We had no control over how the tissue samples were taken in the abattoirs and hence cannot exclude the possibility of cross-contamination between samples at point of collection.

Tissue samples from both VL and NVL samples were initially cut into cubes (5 mm×5 mm) and divided into two visually similar portions in terms of sample size, lesion type and distribution. One portion was processed through the mycobacterial culture procedure and the second portion was frozen at −20°C until prepared for IMS. Samples of NVL only or VL only were processed in separate batches to prevent cross-contamination between potentially heavily infected VL samples and lightly or non-infected NVL samples. Samples were also prepared individually to prevent cross-contamination. All work was carried out in a Class I biological safety cabinet under Containment Level 3 laboratory conditions for health and safety reasons.

### Mycobacterial culture

#### NVL tissue samples

Cubed lymph node sample (approx.10 g) was ground with a small amount of sterile sand using a mortar and pestle before the addition of 12 ml 5% oxalic acid. The sample was mixed again and the liquid content transferred to a centrifuge tube, discarding the remaining solid material from the mortar. The sample was transferred to a rotary mixer and mixed for 15 min at 37°C, followed by centrifugation at 1,620×g for 15 min. The supernatant was discarded and the sediment washed twice in sterile phosphate buffered saline (PBS). Two Lowenstein-Jensen slopes containing pyruvate and two Stonebrink slopes containing pyruvate (Media for Mycobacteria, Cardiff, UK) were inoculated with the resulting sediment using a sterile swab and incubated at 37°C for a minimum of 8 weeks. The remaining sediment (500 µl) in sterile PBS was vortexed to form a liquefied emulsion and inoculated into MGIT (Mycobacterium growth indicator tube) culture medium supplemented with MGIT OADC and MGIT PANTA antibiotic cocktail (both Becton Dickinson) as per the manufacturer's instructions. Each tube was scanned into the Bactec MGIT 960 machine and incubated for up to 8 weeks at 37°C or until growth was detected.

#### VL tissue samples

Approximately 1 g of cubed VL tissue was deposited into a ribolyser tube and 600 µl 5% oxalic acid added. The tissue was homogenised in a Hybaid Ribolyser for 2×30 second cycles at 6.5 m/s, mixed on a rotary mixer for 15 min at 37°C, and then centrifuged at 11,300×g for 5 min. The supernatant was discarded and the sediment neutralised and resuspended by adding 400 µl PBS. Two Lowenstein-Jensen slopes containing pyruvate and two Stonebrink slopes containing pyruvate were inoculated and incubated at 37°C for a minimum of 8 weeks.

#### ‘Lesions at routine slaughter’ tissue samples

The same procedure as for VL samples was applied except that the pellet was neutralised and resuspended using 700 µl PBS, 500 µl of which was inoculated into a MGIT tube and the remainder onto the solid media as above.

### Tissue preparation for Direct PCR and IMS

Cubed lymph node tissue (3 g) was ground with a small amount of sand and 4.5 ml PBS in a sterile mortar. The liquid portion was transferred to a centrifuge tube and the remaining solid material was discarded from the mortar. The sample was centrifuged at 300×g for 3 min to sediment sand and tissue particulates. A 1:10 dilution of the clarified tissue supernatant was made in sterile PBS, 100 µl of which was used directly for IS6110 touchdown PCR and 1 ml for IMS.

#### Direct IS6110 Touchdown PCR

Diluted tissue homogenate (100 µl) was heat-inactivated in screw-capped microcentrifuge tubes by boiling for 25 min at 100°C in a water bath. Samples were centrifuged at 11,300×g for 1 min. DNA was extracted and concentrated from heated samples using Zymoclean DNA Clean and Concentrator^TM^-5 kit (Zymo Research Corporation, Irvine, California, USA) as per kit instructions. DNA was eluted from the column in 10 µl Tris-EDTA buffer (TE, pH 8). Each PCR reaction consisted of 45 µl Platinum Blue PCR SuperMix (Life Technologies) plus 1 µM of each primer (INS1 5′-cgt gag ggc atc gag gtg gc-3′ and INS2 5′-gcg tag gcg tcg gtg aca aa-3′) [13], 2 mM MgCl_2_ and 5 µl of template DNA. Touch-down PCR amplification was performed on a Techne TC-512 thermal cycler according to the protocol used by Zumárraga et al. [14], with an initial denaturation step of 96°C for 3 min, followed by 8 cycles of denaturation at 96°C for 1 min, annealing temperature starting at 72°C for 1 min and decreasing by 1°C/cycle, and 72°C for 1 min for extension. This step was followed by 30 cycles of 96°C for 1 min, 65°C for 1 min, 72°C for 2 min, and a final extension at 72°C for 8 min before holding at 4°C. PCR products were visualised by 2% agarose gel electrophoresis and ethidium bromide staining and gels photographed using UVP gel acquisition software.

#### Automated IMS

Dually coated (Mab 11G3 and biotinylated peptide EEA302) MyOne tosylactivated Dynabeads (10 µl), prepared in-house according to the manufacturer's instructions, were added to the first well of BeadRetriever strip (Life Technologies), 1 ml of PBS-0.05% Tween 20 wash buffer to the next two wells, and 300 µl PBS to the fourth well. The previously prepared 1∶10 dilution (1 ml) of the clarified tissue supernatant was then added to the first well of the BeadRetriever strip. The tube rack, containing up to 15 samples, was transferred to the Dynal BeadRetriever (Life Technologies) and IMS was performed using the pre-loaded ‘Environmental’ programme. This pre-set programme consists of a 35 minute incubation period in well 1 of the bead retriever strip with mixing at a medium speed throughout. The beads are then captured and transferred to well 2 and washed for 1 min at a medium speed. After a second transfer into well 3 the beads are washed again for 1 min at medium speed. The final transfer is into well 4 where the beads are released into 300 µl PBS using a 10 sec high speed mix. The resuspended beads were then split into three 100 µl aliquots for: (1) *M. bovis* touchdown PCR (IMS-PCR); (2) MGIT culture without decontamination (IMS-MGIT) and (3) potential spoligotyping (IMS-spoligotyping).

#### IMS-PCR

DNA was extracted from *M. bovis* cells in 100 µl of each bead suspension by heating in screw-cap microcentrifuge tubes at 100°C for 25 min in a waterbath. After a brief centrifugation, DNA was extracted and concentrated from each sample and IS6110 Touchdown PCR performed as described above for Direct PCR.

#### IMS-MGIT culture

100 µl aliquot of each bead suspension was inoculated directly into a MGIT tube supplemented with MGIT OADC and MGIT PANTA as per the mycobacterial culture procedure (described earlier) and incubated for up to 8 weeks at 37°C. Any IMS-MGIT cultures that indicated growth positive on the MGIT system throughout the incubation period were removed and examined for the presence of acid-fast bacteria typical of *M. bovis* by Ziehl-Neelsen (ZN) staining. Additionally, all IMS-MGIT cultures were subjected to ZN staining at the end of the 8 week incubation period. When acid-fast bacteria were observed a sub-sample of the culture was spoligotyped by the method of Roring et al. [16]. If *M. bovis* typical spoligotype profiles were obtained, cultures were considered *M. bovis* positive, and will be referred to as IMS-MGIT (ZN) positive. Additionally, all IMS-MGIT cultures at the end of the 8 week incubation period were subjected to IS6110 touchdown PCR. Any of these cultures which tested PCR positive were spoligotyped and considered *M. bovis* positive if spoligotyping was successful. These IMS-MGIT results will be referred to as IMS-MGIT (PCR) positive.

#### IMS-spoligotyping

The remaining 100 µl aliquot of bead suspension was stored at −20°C until the IMS-PCR assay indicated a positive result i.e. if there was any evidence of *M. bovis* DNA being present, at which point a selection of IMS-PCR positive samples were thawed, boiled at 100°C for 8 min and then DNA subjected to spoligotyping using the method of Roring et al. [15] which is routinely used at AFBI. In addition, separate trials evaluating the outcome of spoligotyping applied to different concentrations of *M. bovis* AF2122/97 were carried out to assess the likelihood of obtaining a full spoligotype profile if low numbers of *M. bovis* cells were present after IMS. Ten-fold dilutions of *M. bovis* AF2122/97 (containing approx. 5×10^6^–10 CFU/ml) were prepared in PBS, subjected to automated IMS (as described above), and the beads resuspended in 300 µl PBS. A 100 µl aliquot of each sample was boiled for 8 min and spoligotyped [15]. The other 200 µl was boiled for 25 min, DNA extracted and concentrated using the Zymoclean kit, and DNA eluted in 10 µl TE buffer before spoligotyping.

### Statistical analysis of results

Test performance was assessed using McNemar's Chi-Square test (GenStat version 14.2) to analyse 2×2 contingency tables. Survey results were also subjected to generalised linear mixed model analysis (GLMM, Genstat version 14.2) to determine the probability of a positive detection of *M. bovis* by each test and to assess if the type of lymph node tested (VL versus NVL) influenced the test outcome.

## Results and Discussion

### IMS-based methods (IMS-PCR and IMS-MGIT culture) compared to mycobacterial culture and Direct PCR

**Figure 1:**
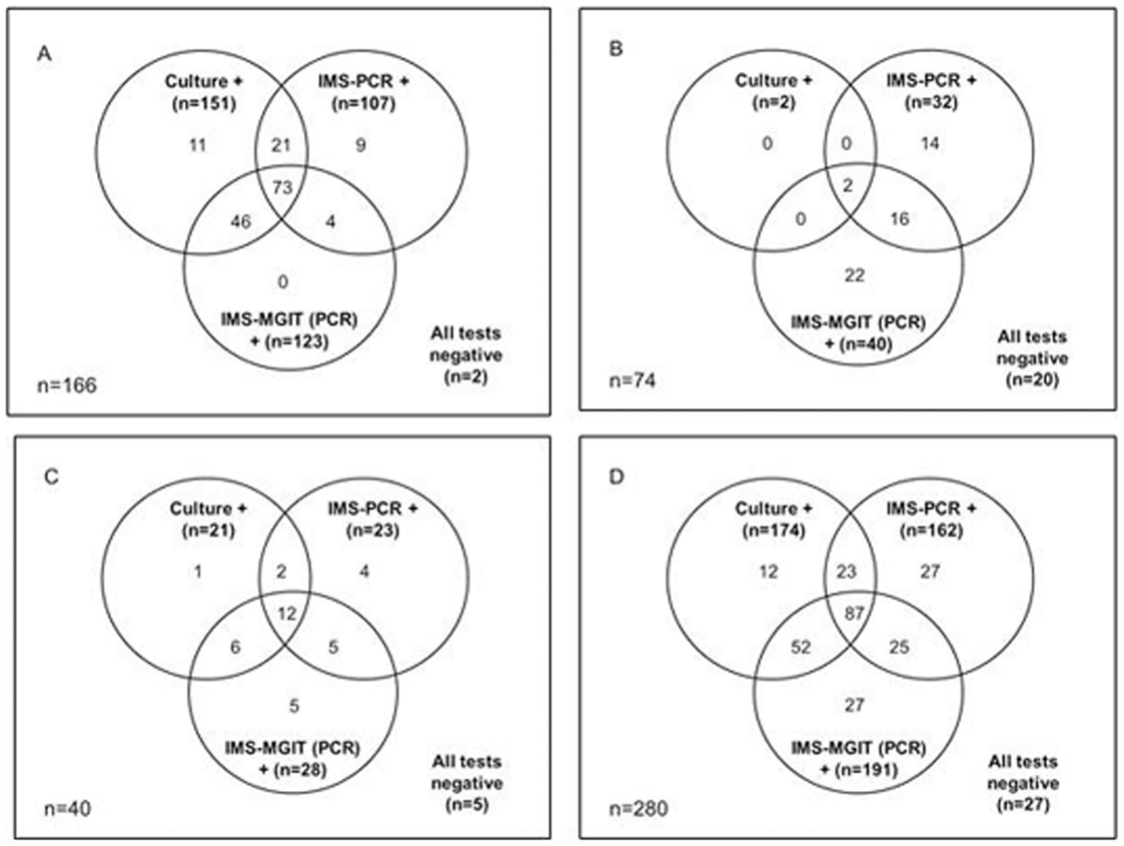
Interrelationships between Culture, IMS-PCR and IMS-MGIT PCR positive results. (A) visibly lesioned lymph nodes from reactor cattle, (B) non-visibly lesioned lymph nodes from reactor cattle, (C) visibly lesioned lymph nodes from non-reactor cattle detected at routine slaughter, and (D) all lymph node samples, irrespective of type.

A total of 280 lymph node tissue samples, comprised of 206 VL samples (166 from skin test reactor animals and and 40 from non-reactor animals with lesions at routine slaughter) and 74 NVL samples, were subjected to culture, Direct PCR, IMS-PCR and IMS-MGIT culture. IMS-PCR, Direct PCR and IMS-MGIT culture were applied to aliquots of the same lymph node homogenate, whereas culture had to be carried out on a separate portion of the same lymph node because it involves initial homogenisation in oxalic acid. Overall, only 27 (9.6%) of the 280 lymph node samples tested were negative for presence of *M. bovis* by all tests applied. In total, 174 lymph node samples –2 (2.7%) of 74 NVL and 172 (83.5%) of 206 VL samples (151 (91.0%) of 166 VL reactors and 21 (52.5%) of 40 VL ‘at routine slaughter’ cases) – tested *M. bovis* positive by the culture method. In the culture system, lymph node culture is only considered positive for *M. bovis* if typical acid-fast cords observed in MGIT cultures or suspect colonies on Lowenstein-Jensen or Stonebrink slopes are subsequently confirmed to be *M. bovis* by spoligotyping. Figure 1 illustrates the interrelationships between culture, IMS-PCR and IMS-MGIT (PCR) results for lymph nodes from reactor animals with VL (Fig. 1A), reactor animals with NVL (Fig. 1B), non-reactor animals with VL at routine slaughter (Fig. 1C), and all lymph node samples irrespective of type (Fig. 1D. Collectively, the IMS-based methods (IMS-PCR and IMS-MGIT culture) detected the presence of *M. bovis* in 162 (93%) of the 174 culture positive samples, but also in an additional 79 (28.2%) lymph node samples (52 NVL and 27 VL samples, comprising 13 from skin test reactor animals and 14 from ‘lesions at routine slaughter’ cases). Hence, the IMS-based methods achieved improved detection rates of *M. bovis* in lymph node tissues, particularly with NVL samples from reactor animals. The majority (112/162, 70%) of the IMS-PCR positive results (result obtained within 48 h of testing) later translated to an IMS-MGIT culture positive result (after 8 week incubation period) confirming the presence of viable *M. bovis* cells in these lymph node samples and not just detection of *M. bovis* DNA. Essentially, the only difference between the IMS-PCR and IMS-MGIT (PCR) results is due to the effect of an 8 week incubation period on captured *M. bovis* cells, which should have permitted resuscitation and replication. However, for 27 of the 78 lymph node samples (14 NVL and 13 VL samples) an initial positive IMS-PCR result did not translate to either a culture or IMS-MGIT (PCR) positive result. These may represent lymph node samples containing *M. bovis* cells that were unable to resuscitate during either culture or IMS-MGIT culture, or dead *M. bovis* cells.

In the case of the NVL samples, the difference between results of culture and IMS-based methods was particularly marked –54 (73%) of 74 NVL samples tested positive for *M. bovis* by IMS-PCR or IMS-MGIT culture, whereas only 2 (2.7%) of 74 NVL samples were recorded as culture positive. This is a particularly important finding and if validated on a larger scale would make a significant contribution to understanding non-visible lesion status, which has confounded comprehension of the disease in cattle. NVL tissues without laboratory disease confirmation are problematic at the epidemiological level [16] since there is no disclosed seat of infection to explain skin test positivity. Confirmation of exposure to *M. bovis* through the presence of specific DNA sequences, or viable *M. bovis*, detected by IMS-based methods would help to explain immune recognition and recall in the absence of culture positive status.

As reported above, an additional 79 lymph nodes were indicated *M. bovis* positive by the IMS-based detection methods (IMS-PCR and IMS-MGIT) than by culture. Amongst this number were 30 IMS-MGIT culture positive lymph nodes emanating from animals in 10 herds from which multiple animals (between two and six animals per herd) had been tested. The animals concerned were principally, but not exclusively, skin test reactor animals presenting with no visible lesions at time of slaughter. When the histories of the 10 herds were further investigated, six of the herds were found to have had a recent history of bTB infection and four were previously bTB free. Therefore, the novel IMS-MGIT culture method was confirming TB infection in these herds, when culture had not. However, it must be acknowledged that interpretation of the enhanced sensitivity of the IMS-based methods may be compromised by the possibility that cross-contamination upon collection of tissue samples from carcases at the abattoir may have occurred. This possibility should be taken into account when interpreting IMS-based method findings and results considered in the context of prior herd and/or animal history.

### Comparison of Direct PCR and IMS-PCR test results with culture results

**Figure 2:**
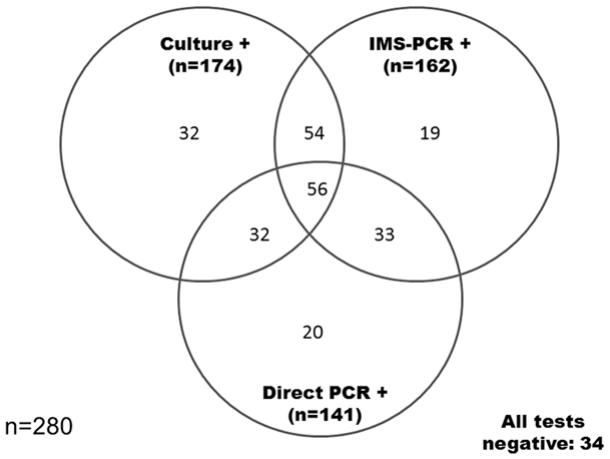
Interrelationships between Culture, Direct PCR and IMS-PCR positive results for all 280 lymph node samples, irrespective of type.

Results of the two PCR detection methods are compared with culture results in Figure 2. PCR bands of variable, but generally low, intensity were observed after IMS-PCR and Direct PCR, which suggested variable numbers of *M. bovis* in PCR positive VL and NVL samples. This would be consistent with the variable numbers of *M. bovis* (486–2.54×10^6^ genome equivalents) estimated to be present in infected lymph node samples by quantitative IS1081 real-time PCR previously [17]. Together, the two IS6110 based PCR methods indicated the presence of MTB complex DNA in 214 (76.4%) of the 280 lymph node samples tested. In contrast, culture indicated the presence of viable *M. bovis* in 174 (62.1%) lymph node samples. Fifty-six (32.2%) of the 174 culture positive samples also gave positive results by both IMS-PCR and Direct PCR. Thirty-two (18.4%) lymph node samples (all VL) tested culture positive for *M. bovis* but tested negative by either IMS-PCR or Direct PCR, or both PCR methods. The remaining 86 (49.4%) culture positive samples also had an IMS-PCR or a Direct PCR positive result. The 32 culture positive but PCR negative results obtained may provide evidence of PCR inhibition, due to the refractory nature of VL lymph nodes, which can be pus-filled, caseated and calcified. Taylor et al. [17] have previously shown that the discrepancy between sensitivity of detection found with purified mycobacterial DNA and direct testing of field samples was due to limited mycobacterial DNA recovery from tissue homogenates rather than PCR inhibition. Further considerations are that: (a) two separate portions of the same lymph node sample were used for culture and PCR methods, whereas the same lymph node homogenate was analysed by the two PCR methods; (b) different amounts of lymph node tissue extract were used in various test protocols; (c) the coated beads have been shown to bind clumps of *M. bovis* cells and as the IMS samples were split before analysis it is possible that low numbers of *M. bovis* were contained in one part of the IMS sample and not the other; and (d) both solid (Lowenstein-Jensen and Stonebrink slopes) and liquid culture media were used during culture (and hence chances of isolating viable *M. bovis* cells may have been higher), whereas only liquid MGIT medium was inoculated after IMS. It is estimated that approximately 0.5 to 2 g was inoculated into MGIT tubes and from 80 mg to 1 g (depending on VL or NVL status) onto slopes of solid culture media during culture, whereas 22.23 mg were used for IMS-MGIT culture, 11.11 mg in an IMS-PCR reaction and 3.35 mg in a Direct PCR reaction. The portion tested by culture may simply have contained more *M. bovis* cells. It subsequently transpired that the sample after IMS had been split into three portions unnecessarily, since IMS-spoligotyping did not prove possible. If IMS samples had been split into two sub-samples (for IMS-PCR and IMS-MGIT culture only) detection sensitivity of these two methods would have increased proportionately.

Surprisingly, results for Direct PCR and IMS-PCR were only in agreement (i.e. samples were positive or negative by both tests) for 55% of lymph node samples tested. Eighty-nine (31.78%) of 280 lymph node samples tested positive by both PCR methods, but overall a higher percentage of lymph node samples tested positive by IMS-PCR (162/280, 57.86%) than by direct PCR (141/280, 50.36%) (Figure 2). The results of the two PCR methods were only significantly associated when results for the different lymph node types were considered separately (McNemar's Chi square test, VL p = 0.032 or NVL p = 0.000, Table 1) but not when VL and NVL results were combined (p = 0.076, Table 1). It must be remembered that the IS6110 PCR target is not confined to *M. bovis* but to MTB complex mycobacteria more generally, so a Direct PCR positive result may not necessarily indicate the presence of *M. bovis* exclusively; although isolation of MTB complex species other than *M. bovis* from cattle in Northern Ireland has been an extremely rare occurrence. In contrast, a PCR positive result after IMS probably does indicate the presence of only *M. bovis*, because of the previously demonstrated specificity of the IMS part of the test [5]. The presence of *M. bovis* DNA in tissue homogenates which would be detected by Direct PCR, but not IMS-PCR, and the fact that IS6110 homologs from non-MTB complex bacteria have previously been reported [12], [13], are also possible explanations for difference between Direct PCR and IMS-PCR findings.

**Table 1:** Comparison of IMS-PCR and Direct PCR results for 280 lymph node samples –74 non-visibly lesioned (NVL) and 206 visibly lesioned (VL).

Type of sample (No.)	IMS-PCR+, Direct PCR +	IMS-PCR +, Direct PCR −	IMS-PCR −, Direct PCR +	IMS-PCR −, Direct PCR −	χ^2^ (*P* value.^a^)
VL (206)	71	59	36	40	4.594 (0.032)
NVL (74)	18	14	17	25	0.000 (0.000)
All (280)^b^	89	73	53	65	3.150 (0.076)

a Determined by McNemar's Chi-Square test using Genstat version 14.2.

b Includes 166 VL and 74 NVL samples from TB skin test reactor animals plus 40 VL samples from non-reactor animals detected at routine slaughter.

Data represent number of samples with each combination of test results.

Estimates of the detection sensitivity and specificity of the alternative detection methods relative to culture (the gold standard method [4], although acknowledged as imperfect) for each lymph node type were calculated. Test specificity is regarded as the probability than an uninfected animal will test negative, while test sensitivity is defined as the probability that a truly infected animal will test positive [16]. When NVL lymph nodes were tested the sensitivity of both Direct PCR and IMS-PCR was 100% (95% CI: 15.81–100.0%) and specificities were 55.56% (95% CI: 43.38–67.29%) and 56.94% (95% CI: 46.13–69.81%), respectively. When VL lymph nodes were tested, the sensitivities of Direct PCR and IMS-PCR were 50.00% (95% CI: 42.31–57.69%) and 62.21% (95% CI: 54.46–69.49%), respectively, and the specificities were lower at 38.24% (95% CI: 22.17–56.41%) and 35.29% (95% CI: 19.74–53.53%), respectively. However, as culture is considered the reference method for statistical analysis, the consequence of extra test positives with IMS-PCR and Direct PCR relative to culture is that these calculated values for specificity are lower than they would actually be in reality [18]. This finding of greater detection sensitivity and specificity with IMS-PCR compared to direct PCR is consistent with those of previous studies evaluating the impact of IMS before PCR on detection of *M. bovis* in tissue or environmental samples [19], [20], [21].

### Comparison of results of all detection methods

The performance of all alternative test methods relative to culture was assessed by statistical analysis using McNemar's Chi-Square test to analyse 2×2 contingency tables. The outcomes for all 280 lymph node samples, irrespective of lymph node type, are summarised in Table 2. Results for the IMS-based methods (except IMS-MGIT culture checked by ZN staining at end of incubation period) were not significantly associated with culture results (McNemar's Chi-square test, p = 0.311 for IMS-PCR and p = 0.106 for IMS-MGIT culture confirmed by IS6110 PCR at end of incubation period), whereas Direct PCR and IMS-MGIT culture confirmed by ZN staining at end of incubation period were significantly associated (McNemar's Chi-square test, p = 0.007 and p = 0.000, respectively).

**Table 2:** Comparison of results for alternative detection methods and culture (MGIT broth or Lowenstein-Jensen slope).

Alternative method and result	Culture result		χ^2^ (P value^a^)
	Positive	Negative	
Direct PCR +	88	53	7.367 (0.007)
Direct PCR –	86	53	
IMS-PCR +	109	53	1.025 (0.311)
IMS-PCR –	65	53	
IMS-MGIT (ZN) +	59	4	101.681 (0.000)
IMS-MGIT (ZN) –	115	102	
IMS-MGIT (PCR) +	139	51	2.616 (0.106)
IMS-MGIT (PCR) –	35	55	

a Determined by McNemar's Chi-Square test using GenStat Release 14.2.

Results for all 280 lymph node samples are not broken down by lymph node type.

**Figure 3:**
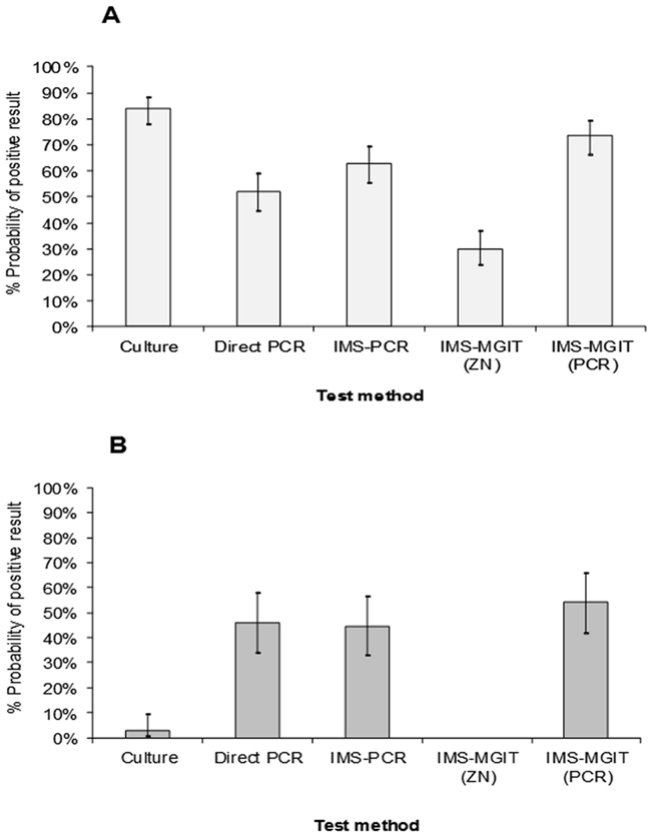
Relative probabilities of *M. bovis* detection in (A) visibly lesioned and (B) non-visibly lesioned lymph nodes by culture and four alternative detection methods. Three of the alternative methods employ IMS as first step.

Test results were also subjected to generalised linear mixed model analysis (GLMM) to determine the probability of a positive detection of *M. bovis* by each test, and to assess if the type of lymph node tested (VL v NVL) influenced the test outcome. GLMM analysis indicated that there was a significant effect of lymph node type per se (p<0.001) on probability of a positive test outcome, which is not really surprising given that VL samples are likely to contain higher numbers of *M. bovis* cells than NVL samples and so less detection sensitivity would be required of a testing method to achieve a positive result. GLMM analysis also indicated a significant interaction between lymph node type and test outcome (p<0.001) in terms of *M. bovis* detection, so each test method did not have the same probability of yielding a positive result for the two sample types. For this reason, probabilities of *M. bovis* detection by each test are presented separately for VL and NVL lymph nodes samples in Figure 3. It was clear from these results that in the case of NVL tissues three of the alternative detection methods (Direct PCR, IMS-PCR and IMS-MGIT culture (with PCR and spoligotyping confirmation)) had higher probabilities (45.94%, 44.59% and 54.14%, respectively) of detecting presence of *M. bovis* than culture (2.63%). In contrast, for VL lymph node testing the highest probability of a positive test result was obtained with culture (83.83%) and IMS-MGIT culture (with PCR and spoligotyping confirmation of the presence of *M. bovis*) was best of alternative tests (73.13%).

On the basis of the culture results obtained during this study, our assessment is that the current culture protocol is deficient in two areas. Firstly, oxalic acid treatment is employed to decontaminate the lymph node sample in order to inactivate non-mycobacterial contaminants before culture, despite the knowledge that some inactivation of *M. bovis* cells will occur. Use of a detergent, alkali or acid treatment for decontamination of lymph node tissue for *M. bovis* isolation is the prescribed method [4], so use of oxalic acid is not unique to bTB testing in Northern Ireland. However, the impact of its use is that *M. bovis* cells present in NVL lymph nodes in low numbers are not being detected routinely by culture, which undoubtedly has implications for the confirmation of *M. bovis* in tissue samples. Secondly, ZN staining is routinely used to confirm the presence of, typical acid-fast cells in MGIT culture positive samples before spoligotyping; PCR confirmation of the presence/absence of *M. bovis* DNA in MGIT cultures is not currently undertaken. This study showed that confirmation of the presence of *M. bovis* in IMS-MGIT cultures by ZN staining is a very insensitive method; sensitivities of 0.00% for NVL and 34.3% for VL samples relative to culture results. However, both ZN staining and PCR testing of IMS-MGIT cultures was performed, and results clearly indicate that a significant number of *M. bovis* positive MGIT cultures would have been reported as negative solely on the basis of ZN staining, when in reality low numbers of slowly growing, resuscitating *M. bovis* cells may be present.

### Spoligotyping

The potential to apply spoligotyping directly after immunomagnetic capture of *M. bovis* from lymph node material was investigated because if it were possible this would allow earlier confirmation of the presence of *M. bovis* infection in lymph nodes. In preliminary trials on a range of dilutions of *M. bovis* AF2122/96, a full spoligotype profile was only obtained after IMS and Zymoclean concentration when ≥5×10^3^ CFU/ml were present before IMS; partial or no spoligotype profiles were obtained otherwise. When a 100 µl aliquot of beads from a selection of IMS-PCR positive lymph node samples was subjected to spoligotyping, full spoligotype profiles were never obtained. The spoligotyping protocol was originally optimised for DNA from *M. bovis* cultures [15], which would contain a much higher quantity of cells, and hence DNA, than obtained from cells captured from lymph nodes by IMS. Therefore, spoligotyping directly after IMS was not considered to be a feasible method for quickly demonstrating the presence of *M. bovis* in a lymph node sample.

**Figure 4:**
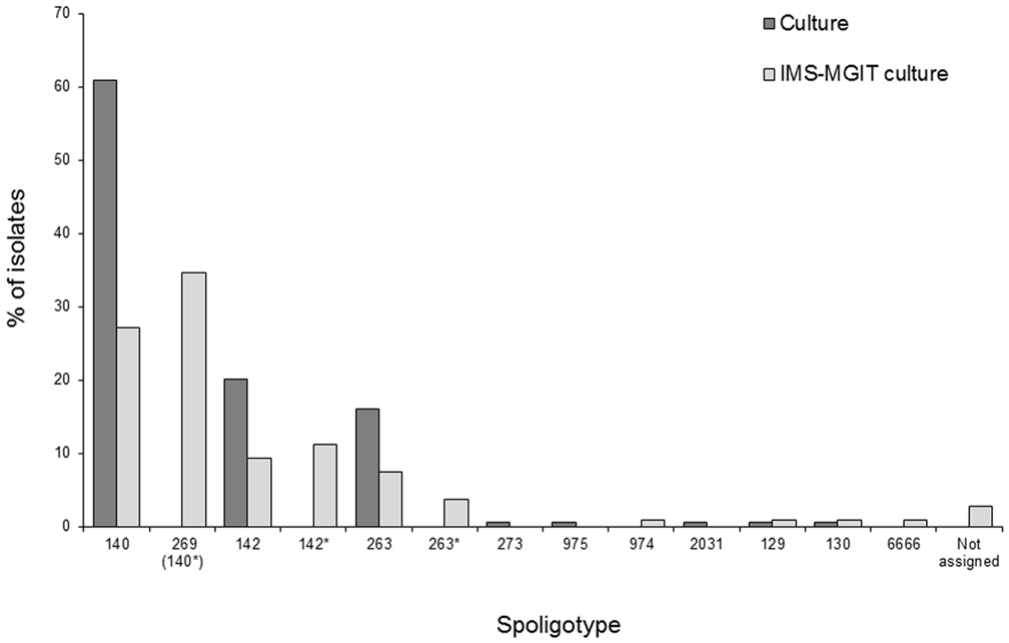
Distribution of *M. bovis* spoligotypes isolated by culture and IMS-MGIT culture. Spoligotypes 269 (140*), 142* and 263* are thought to be spoligotypes 140, 142 and 263 with spacer 15 signal absent which have arisen due to lesser amounts of *M. bovis* DNA being present in IMS-MGIT cultures.

Spoligotyping was used in the usual manner to confirm the isolation of *M. bovis* by testing slope or MGIT cultures. The presence of *M. bovis* was confirmed in all 174 positive cultures and in 107 of 190 positive IMS-MGIT (PCR) cultures by spoligotyping. The remaining 83 IMS-MGIT (PCR) positive cultures were not subjected to spoligotyping because the earlier experiments had indicated that high numbers of *M. bovis* were needed to yield sufficient DNA to ensure successful spoligotyping, and for these IMS-MGIT culture samples the PCR positive results generally indicated the presence of low numbers of *M. bovis*/low levels of *M. bovis* DNA. The distribution of spoligotypes obtained for cultures and IMS-MGIT cultures is shown in Figure 4. The range of different *M. bovis* spoligotypes isolated by the IMS-MGIT culture method was not appreciably different from the range isolated by culture, which was reassuring considering that the antibody and peptide on the magnetic beads were generated using a single *M. bovis* spoligotype (SB140). However, a consistent observation for many of the spoligotypes obtained from IMS-MGIT cultures was the absence of a signal indicating spacer 15, meaning that spoligotypes that were probably a recognised spoligotype (SB140, SB142 or SB263) were reported as SB269, SB142* and SB263*, respectively. Interestingly, in the case of the spoligotypes from IMS-MGIT cultures, if the number of spacer 15-containing and corresponding spacer 15-deficient cultures is combined (i.e. SB140+ SB269, SB142+ SB142* and SB263+ SB263*) then the percentages of each spoligotype for IMS-MGIT cultures almost matches percentages for SB140, SB142 and SB263 spoligotypes for cultures and both would then be consistent with reported *M. bovis* spoligotype distributions in Northern Ireland cattle [22]. Therefore, this suggests that the spoligotyping results obtained are probably due to borderline amounts of *M. bovis* DNA being extracted from the IMS-MGIT cultures. The influence of DNA quantity on spoligotype obtained has been demonstrated for *M. bovis* AF2122/97 in the course of this study (when DNA was extracted from a sample containing <5×10^3^ cells/ml, spoligotype SB269 was reported instead of 140, due to lack of signal corresponding to spacer 15) but merits confirmation with *M. bovis* spoligotypes SB142 and SB263, and perhaps other spoligotypes also.

## Conclusions

In this study there is evidence of higher *M. bovis* detection rates using the IMS-based methods (IMS-PCR and IMS-MGIT culture) compared to conventional culture, notably with bTB skin test reactor animals presenting with non-visible lesions. Significantly, using IMS-based methods, viable *M. bovis* cells were recovered from NVL lymph node samples taken from skin test positive animals, demonstrating *M. bovis* when culture methods failed to do so. Using IMS to retrieve *M. bovis* from tissues circumvents the need to decontaminate prior to culture and it is likely that this has a positive impact on cell viability and hence improved recovery rates from clinical samples.

There is potential to improve detection of *M. bovis* from clinical specimens using IMS as a novel first step in sample preparation. This method introduces a high degree of specificity for *M. bovis* by selecting cells in apparently different physiological states (viable and non-viable). The range of *M. bovis* spoligotypes isolated by the IMS-MGIT culture method was very similar to the range isolated by culture, implying that IMS can select a very broad range of spoligotypes.

Confirmation of *M. bovis* in clinical samples can be streamlined through the preparation of a single IMS treated sample which is then split into two aliquots. PCR would be applied to the first aliquot to rapidly detect *M. bovis* infected tissues within 24–48 hours. MGIT culture would be applied to the second aliquot and after 8 weeks incubation all cultures would be subjected to PCR (targeting IS6110 or other suitable target) to confirm the presence of viable *M. bovis.* Increased detection sensitivity by the use of PCR to confirm the presence of *M. bovis* in MGIT cultures compared with ZN staining was indicated by this study.
